# RIP3 impedes transcription factor EB to suppress autophagic degradation in septic acute kidney injury

**DOI:** 10.1038/s41419-021-03865-8

**Published:** 2021-06-08

**Authors:** Ruizhao Li, Xingchen Zhao, Shu Zhang, Wei Dong, Li Zhang, Yuanhan Chen, Zhilian Li, Huan Yang, Ying Huang, Zhiyong Xie, Weidong Wang, Chunling Li, Zhiming Ye, Zheng Dong, Xinling Liang

**Affiliations:** 1 Division of Nephrology, Guangdong Provincial People’s Hospital, Guangdong Academy of Medical Sciences, Guangzhou, Guangdong China; 2 Department of Nephrology, Sun Yat-sen Memorial Hospital, Sun Yat-sen University, Guangzhou, Guangdong China; 3 The Second School of Clinical Medicine, Southern Medical University, Guangzhou, China; 4 Institute of Hypertension, Zhongshan School of Medicine, Sun Yat-sen University, Guangzhou, Guangdong China; 5 Department of Cellular Biology and Anatomy, Medical College of Georgia at Augusta University and Charlie Norwood Veterans Affairs Medical Center, Augusta, GA USA

**Keywords:** Macroautophagy, Acute kidney injury

## Abstract

Autophagy is an important renal-protective mechanism in septic acute kidney injury (AKI). Receptor interacting protein kinase 3 (RIP3) has been implicated in the renal tubular injury and renal dysfunction during septic AKI. Here we investigated the role and mechanism of RIP3 on autophagy in septic AKI. We showed an activation of RIP3, accompanied by an accumulation of the autophagosome marker LC3II and the autophagic substrate p62, in the kidneys of lipopolysaccharide (LPS)-induced septic AKI mice and LPS-treated cultured renal proximal tubular epithelial cells (PTECs). The lysosome inhibitor did not further increase the levels of LCII or p62 in LPS-treated PTECs. Moreover, inhibition of RIP3 attenuated the aberrant accumulation of LC3II and p62 under LPS treatment in vivo and in vitro. By utilizing mCherry-GFP-LC3 autophagy reporter mice in vivo and PTECs overexpression mRFP-GFP-LC3 in vitro, we observed that inhibition of RIP3 restored the formation of autolysosomes and eliminated the accumulated autophagosomes under LPS treatment. These results indicated that RIP3 impaired autophagic degradation, contributing to the accumulation of autophagosomes. Mechanistically, the nuclear translocation of transcription factor EB (TFEB), a master regulator of the lysosome and autophagy pathway, was inhibited in LPS-induced mice and LPS-treated PTECs. Inhibition of RIP3 restored the nuclear translocation of TFEB in vivo and in vitro. Co-immunoprecipitation further showed an interaction of RIP3 and TFEB in LPS-treated PTECs. Also, the expression of LAMP1 and cathepsin B, two potential target genes of TFEB involved in lysosome function, were decreased under LPS treatment in vivo and in vitro, and this decrease was rescued by inhibiting RIP3. Finally, overexpression of TFEB restored the autophagic degradation in LPS-treated PTECs. Together, the present study has identified a pivotal role of RIP3 in suppressing autophagic degradation through impeding the TFEB-lysosome pathway in septic AKI, providing potential therapeutic targets for the prevention and treatment of septic AKI.

## Introduction

Sepsis is the leading cause of acute kidney injury (AKI) in critically ill patients, characterized by a rapid decrease of renal function accompanied by multiorgan dysfunction syndromes. Septic AKI is associated with high morbidity, accounting for nearly 50% of AKI cases in critically ill patients, and high mortality reaching up to 60–70%^1–5^. Pathologically, one of the major features of septic AKI is sublethal and lethal injury of renal tubular cells^6^. However, the mechanisms underlying the pathophysiology of septic AKI are poorly understood.

Receptor interacting protein kinase 3 (RIP3) is a serine/threonine kinase that plays a crucial role in mediating inflammation and cell death^7^. RIP3 was activated in the renal tubules of septic AKI mice and lipopolysaccharide (LPS)-treated cultured proximal tubular epithelial cells (PTECs)^8^. Our recent study showed that a RIP3-selective inhibitor attenuated renal tubular apoptosis and renal dysfunction in LPS-induced septic AKI mice^9^. Sureshbabu et al.^8^, using proximal tubule-specific RIP3-knockout mice, showed an alleviation of tubular injury and restoration of renal function in septic AKI mice, further demonstrating the pathogenetic role of RIP3 in septic AKI. However, it is still unknown how RIP3 mediates nephrotoxicity and how RIP3 inhibitors protect the kidneys during septic AKI. Our previous study suggested RIP1 inhibited autophagy in LPS-induced cultured renal tubular cells^10^. In this study, we aimed to investigate the role of RIP3 in regulating autophagy during septic AKI.

Autophagy is a highly conserved process that degrades and recycles defective proteins and organelles via lysosomes to maintain cellular homeostasis^11–13^. The dynamic process of autophagy is called “autophagic flux,” mainly including the formation of autophagosomes, fusion of autophagosomes and lysosomes to autolysosomes, and degradation of substrates in autolysosomes^12^. Autophagy dysfunction is associated with the pathogenesis of a variety of diseases^14–16^. Using pharmacological regulators of autophagy and autophagy-deficient animal and cell models, studies have demonstrated a pivotal renal-protective role of autophagy in septic AKI^10,17–21^. However, the mechanism regulating autophagy during septic AKI remains unclear.

Transcription factor EB (TFEB), a “master regulator” of both the lysosome and autophagy pathway, drives the transcription of multiple lysosome- and autophagy-related genes involved in lysosome biogenesis and degradation, as well as most of the steps of autophagic flux from autophagosome formation to degradation of substrates in autolysosomes^22,23^. The activity of TFEB depends largely on its subcellular distribution, which is activated to translocate to the nucleus while inactivated to be retained in the cytoplasm, modified by its phosphorylation through several serine threonine kinases^24^. There is a significant lack of understanding of the role of TFEB in renal tubules and septic AKI.

Based on these findings and questions, the present study aimed to investigate the role and underlying mechanism of RIP3 in regulating autophagy in renal tubular cells and septic AKI.

## Materials and methods

### Ethics statement and patient samples

The protocol of this study was approved by the Ethics Committee of Guangdong Provincial People’s Hospital (GDREC2016258H; Guangdong, China). The patient study was conducted according to the Helsinki Declaration. Human kidney samples were obtained from one patient diagnosed with septic AKI and one patient diagnosed with minimal change disease, who underwent the renal biopsies at the Guangdong Provincial People’s Hospital with the informed written consent of these patients. Human kidney samples obtained from the patient with minimal change disease were used as control, which were confirmed to be normal renal tissue by further pathological examination. Clinical characteristics of the patient with septic AKI and control subject are presented in the Supplementary Table 1.

### Animals

All animal procedures undertaken according to the protocols of the Guangdong Academy of Medical Sciences and were approved by the Ethics Committee of Guangdong Provincial People’s Hospital. Mice were housed in a pathogen-free animal facility of Guangzhou Forevergen Biosciences (Guangzhou, Guangdong, China) under a 12 h light/12 h dark cycle with free access to food and water. C57BL/6 mice were purchased from Nanjing Biomedical Research Institute of Nanjing University (Nanjing, Jiangsu, China). The Ksp1.3/Cre transgenic mice (Ksp-Cre) were purchased from Jackson Laboratory (012237; Jackson Labs, Bar Harbor, ME). The renal tubular cell-specific mCherry-green fluorescent protein (GFP)-LC3 autophagy reporter mice (R26-LSL-mCherry-GFP-LC3/R26-LSL-mCherry-GFP-LC3; Ksp-Cre) were generated by Shanghai Model Organisms Center, Inc. (Shanghai, China). Briefly, the Cre recombinase-conditional mCherry-GFP-LC3 knock-in mouse model (R26-LSL-mCherry-GFP-LC3/+) was crossed with the Ksp-Cre mice (Supplementary Fig. 1). The mCherry-GFP-LC3 autophagy reporter mice was used to test autophagic flux as previously described^25,26^. The mCherry-GFP-LC3 protein initially recruited to the membranes of autophagosomes upon the induction of autophagy and was then delivered to autolysosomes. The GFP signal is sensitive to and quenched by the acidic conditions within the lysosomes, but the mCherry fluorescent signal is stable in the lysosome. Colocalization of GFP and mCherry signals (yellow fluorescence) indicates autophagosomes that have not yet fused with lysosomes. The mCherry signal alone without GFP signal (red-only fluorescence) indicates autolysosomes.

### LPS-induced septic AKI mouse model

The 8- to 12-week-old male C57BL/6 mice or mCherry-GFP-LC3 reporter mice were randomly divided into three groups: (i) LPS group, mice were injected with LPS (10 mg/kg, intraperitoneal (i.p.) injection; MilliporeSigma, Burlington, MA, USA); (ii) LPS + GSK’872 group, mice were injected with the RIP3-selective inhibitor GSK’872 (5 μM/kg, i.p. injection; Selleck Chemicals, Houston, TX, USA) 15 min prior to LPS treatment (10 mg/kg, i.p. injection); and (iii) control group, mice were injected with sterile saline (i.p. injection). Blood samples were collected at 6, 12, 24, 48, and 72 h after LPS administration. Mice were sacrificed at 6, 12, 24, 48, and 72 h after LPS treatment for histological and biochemical analyses.

### Cell culture and treatment

Mouse primary renal PTECs (C57-6015; Cell Biologics, Chicago, IL, USA) were cultured in Dulbecco’s modified Eagle’s medium/F12 medium (Thermo Fisher Scientific, Waltham, MA, USA) supplemented with 10% fetal bovine serum (Thermo Fisher Scientific) as described previously^9^. Cells were treated with LPS (10 μg/mL; MilliporeSigma) for 3, 6, 12, 24, and 48 h. For RIP3 inhibition, cells were treated with GSK’872 (0.3 μM; Selleck Chemicals) 1 h prior to LPS treatment (10 μg/mL) for different time points as indicated. Control cells were treated with an equal volume dimethyl sulfoxide. To study autophagic flux, cells were treated with chloroquine (CQ; 20 μM; MilliporeSigma) for 24 h. The autophagy flux was determined by comparing the levels of LC3II and p62/SQSTM1 in the presence vs. absence of CQ.

### Transfection of adenovirus and siRNAs

For RIP3, TFEB knockdown, cells were transfected with 50 nM RIP3 small interfering RNA (siRNA) or 50 nM TFEB siRNA (RiboBio, Guangzhou, Guangdong, China) for 12–48 h, using Lipofectamine 2000 (Thermo Fisher Scientific, Scotts Valley, CA, USA) according to the manufacturer’s protocol. For mixed-lineage kinase domain-like protein (MLKL) knockdown, cells were transfected with 50 nM MLKL siRNAs with three different sequences (MLKL siRNA 1–3). MLKL siRNA-3 significantly decreased the protein expression of MLKL and was used for the subsequent experiments. A scrambled siRNA (RiboBio) was used as control. The sequences of the RIP3 siRNA, TFEB siRNA, and MLKL siRNAs were listed in the Supplementary Table 2. For TFEB overexpression, cells were transfected with TFEB-overexpression plasmid (Hanbio Technology, Shanghai, China) or its vector plasmid as control for 12–48 h, using Lipofectamine 2000. To investigate the autophagic flux, cells were transfected with monomeric red fluorescent protein (mRFP)-GFP-LC3 adenovirus (Hanbio Technology) as we described previously^27^. The mRFP-GFP-LC3 protein initially recruited to the membranes of autophagosomes upon the induction of autophagy and was then delivered to autolysosomes. The GFP fluorescent signal is sensitive to and quenched by the acidic conditions within the lysosomes, but the mRFP fluorescent signal is stable in the lysosome. Colocalization of GFP and mRFP signals (yellow fluorescence) indicates autophagosomes that have not yet fused with lysosomes. The mRFP signal alone without the GFP signal (red-only fluorescence) indicates autolysosomes.

### Antibodies

The following primary antibodies were used for immunoblot analysis: anti-β-actin antibody (1:1000 dilution; catalog number 8457; Cell Signaling Technology, Danvers, MA, USA), anti-Histone H3 (1:1000 dilution; catalog number AF0863; Affinity Biosciences, Cincinnati, OH, USA), anti-LC3A/B antibody (1:1000 dilution; catalog number 4108; Cell Signaling Technology), anti-p62 antibody (1:1000 dilution; catalog number 5114; Cell Signaling Technology), anti-RIP3 antibody (1:1000 dilution; catalog number ab56164; Abcam, Cambridge, UK), anti-phosphorylated RIP3 (p-RIP3) antibody (1:1000 dilution; catalog number ab195117; Abcam), anti-TFEB antibody (1:1000 dilution; catalog number A303-673A; Bethyl, Montgomery, TX, USA), anti-Cathepsin B (CTSB) antibody (1:1000 dilution; catalog number ab58802; Abcam), anti-LAMP1 antibody (1:1000 dilution; catalog number ab25630; Abcam), and anti-MLKL antibody (1:1000 dilution; catalog number 37705; Cell Signaling Technology). The following primary antibodies were used for Immunofluorescence staining: anti-TFEB antibody (1:100 dilution; catalog number LS-B5907; LifeSpan BioSciences, Seattle, WA, USA), anti-RIP3 antibody (1:1000 dilution; catalog number ab56164; Abcam), anti-LAMP1 (1:200 dilution; catalog number ab24170; Abcam), and anti-CTSB antibody (1:200 dilution; catalog number ab58802; Abcam).

### Subcellular protein fractionation and immunoblotting

Total protein was extracted from kidney tissue or cultured proximal renal tubular cells using radioimmunoprecipitation assay (RIPA) lysate buffer (Beyotime Biotechnology, Shanghai, China) supplemented with protease inhibitor cocktail tablets (Roche, Basel, Switzerland) or protease inhibitor mixture (Beyotime Biotechnology), respectively. Nuclear protein was extracted according to the manufacturer’s protocol of CelLytic^TM^ NuCLEAR^TM^ Extraction Kit (MilliporeSigma, Burlington, MA, USA). Protein concentration was detected using a BCA protein assay kit (Thermo Fisher Scientific, Waltham, MA, USA). The protein of extracts was subjected to immunoblotting analysis using the method described previously^9^. Histone was used as the loading control of the nuclear protein^28^. β-actin were used as the loading control of the total protein.

### Measurement of serum creatinine and BUN

Renal function was assessed by testing serum creatinine and blood urea nitrogen (BUN) using QuantiChrom^TM^ Creatinine Assay kit and QuantiChrom^TM^ Urea Assay kit (BioAssay Systems, Hayward, CA, USA), respectively.

### Histological analysis

Paraffin-embedded kidney tissues were cut into 4 μm sections and fixed with 4% paraformaldehyde and stained with hematoxylin/eosin or periodic acid–Schiff as described previously^9,27^. Tubular injury score was calculated by the area of tubular epithelial cell vacuolar deformation, loss of brush border, tubular dilation, cast formation, and cell lysis. Scores 0, 1, 2, 3, and 4 represented the area of injury <10%, 10–25%, 25–50%, 50–75%, and >75% of the total area, respectively. At least six different fields under the microscope (×400) were randomly selected and an average score was calculated. Histopathology was evaluated by two pathologists in a double-blinded manner.

### Reverse-transcription quantitative PCR

Total RNA was extracted from the cultured cells as described previously^9^. Briefly, cells were treated with TRIzol reagent (Thermo Fisher Scientific) complying with the manufacturer’s instructions and then reversed to cDNAs using a PrimeScript RT Reagent Kit (Takara, Kyoto, Japan). The cDNAs were used for quantitative PCR using a Power SYBR Green PCR Master Mix (TaKaRa). Data were analyzed by the 2^−ΔΔCq^ method with glyceraldehyde 3-phosphate dehydrogenase as the internal control. Primer sequences are listed in the Supplementary Table 3.

### Immunofluorescence staining

Coverslips covered with cells or frozen tissue sections were fixed in 4% paraformaldehyde for 15 min at room temperature. After washing in PBS, cells or frozen tissue sections were permeabilized with 0.1% Triton X-100 in PBS for 5–10 min at room temperature and then blocked with 5% bovine serum albumin for 30 min. Cells or frozen tissue sections were incubated with the primary antibody overnight at 4 °C. The cells or tissue sections were incubated in the dark with donkey anti-goat IgG(H + L) 488 (catalog number A-11055; Invitrogen, Carlsbad, CA, USA) and donkey anti-rabbit IgG(H + L) 555 (catalog number A-31572; Thermo Fisher Scientific) at room temperature for 1 h and then cell nuclei were stained with 4′,6-diamidino-2-phenylindole solution (MilliporeSigma) for 5 min. The slides were imaged by a Nikon Eclipse Ni-E confocal laser scanning microscope (Nikon, Tokyo, Japan) and analyzed with Image-Pro Plus v6.0 (Media Cybernetics, Rockville, MD, USA).

### Co-immunoprecipitation

Co-immunoprecipitation (CoIP) experiments were undertaken using a Dynabeads Protein G Immunoprecipitation kit (Thermo Fisher Scientific) according to the manufacturer’s instructions. Briefly, cell lysates were extracted from culture cells using Pierce IP Lysis Buffer (87787; Thermo Fisher Scientific) supplemented with protease inhibitor (Beyotime Biotechnology). After removing nonspecific binding by Dynabeads Protein G, the cell lysates were added with an appropriate amount of anti-TFEB antibody (catalog number A303-673A; Bethyl) or anti-RIP3 antibody (catalog number ab56164; Abcam), or control IgG and then incubated at 4 °C overnight. The Dynabeads-Ab-antigen complex was washed with washing buffer and eluted with RIPA buffer for western blotting.

### Chromatin immunoprecipitation

The chromatin immunoprecipitation (ChIP) assay was performed according to the manufacturer’s protocol (MilliporeSigma). Briefly, cells were fixed with formaldehyde to fix proteins to DNA. DNA–protein fragments were pulled down with anti-TFEB antibody (ab2636; Abcam). The DNA–protein cross-link was reversed and the DNA was purified. The enrichment of particular DNA sequences was detected by downstream real-time PCR analysis. Primer sequences were listed in the Supplementary Table 4.

### Electron microscopy

Renal tissues from mice were prepared as described previously^9,10^. Briefly, renal tissues were fixed with 2.5% glutaraldehyde, 4% paraformaldehyde, and 0.02% picric acid in 0.1 M cacodylate buffer (pH 7.2) overnight at 4 °C. After washing with cacodylate buffer, renal tissues were post-fixed with 1% osmium tetroxide and 1.5% potassium ferrocyanide for 2 h, dehydrated by a graded series of ethanol (50%, 70%, 80%, 90%, and 100%) and 100% propylene oxide, and then embedded in resin (Epon 812) for 12 h. The ultrastructure was observed using a transmission electron microscope (TEM; JEM-100CX; JEOL, Ltd., Tokyo, Japan). The initial autophagic vacuoles, which indicated autophagosomes, were identified by a double-membrane structure, which was usually at least partly visible as two parallel membrane bilayers separated by an electron-lucent cleft and its morphologically intact contents. The late autophagic vacuoles, which indicated autolysosomes, were identified by a single-membrane structure and its electron-dense contents at various stages of degradation^29^.

### Statistical analysis

Results are presented as mean ± SEM of at least three independent experiments. For animal studies, sample size was estimated according to our previous studies^9^, which performed similar experiments to detect significant difference between samples. Estimations of the minimal sample size for the variables measured in this study were assessed by power analysis, with an *α*-level of 0.05 and a power of 0.8, using StatMate 2 (http://graphpad.com/scientific-software/statmate/). Statistical analysis of the data was conducted using SPSS 21.0. Statistical differences between two groups were determined by Student’s *t*-test. Statistical differences among multiple groups were determined by one-way analysis of variance followed by Fisher’s least significant difference post hoc test. The variance was similar between groups that were being statistically compared. *P* < 0.05 was considered a statistically significant difference.

## Results

### RIP3 was activated to suppress autophagic degradation and lead to tubular injury and renal dysfunction in septic AKI mice

**Fig. 1:**
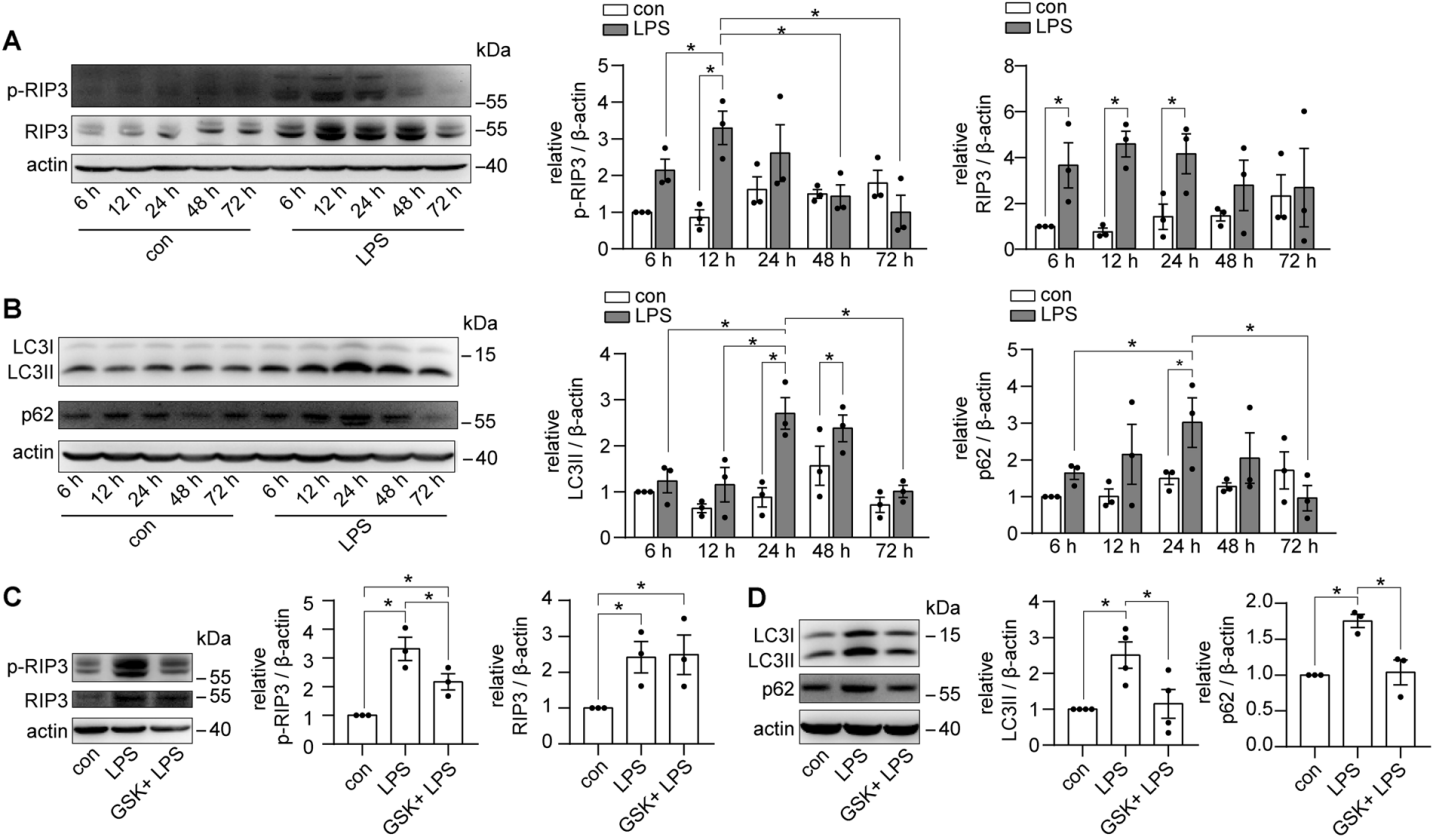
RIP3 was activated to inhibit autophagy in septic AKI mice. **A** Kidney tissues were collected from C57BL/6 mice treated with LPS or sterilized saline (con) for immunoblot of phosphorylated RIP3 (p-RIP3, a marker of RIP3 activity) and total RIP3. p-RIP3 and RIP3 expression, against β-actin expression, were increased in the kidneys of LPS-induced mice (*n* = 3). **B** Immunoblot showed an accumulation of the expression of LC3II (marker for autophagosome) and p62 (substrate for autophagy) in the kidneys of LPS-induced mice (*n* = 3). **C** C57BL/6 mice were treated with sterilized saline (con), LPS, or LPS plus GSK’872 (GSK) for 12 h. Immunoblotting showed that GSK attenuated the increased p-RIP3 in the kidneys of LPS-induced mice (*n* = 3). **D** C57BL/6 mice were treated with sterilized saline (con), LPS, or LPS plus GSK for 24 h. Immunoblot showed that GSK attenuated the accumulated LC3II and p62 in the kidneys of LPS-induced mice (*n* = 3–4). *P < 0.05.

The activity of RIP3 was enhanced significantly in the kidney tissues of LPS-induced septic AKI mice, with a peak at 12 h of LPS treatment (Fig. 1A). Along with the RIP3 activation, the level of the autophagosome marker LC3II was increased at 24 and 48 h of LPS treatment (Fig. 1B), indicating an accumulation of autophagosomes, which could be caused by an increase of autophagosome formation or a blockage of autophagic degradation. In addition, p62, a substrate for autophagy, was accumulated at 24 h of LPS treatment (Fig. 1B), suggesting a suppression of autophagic degradation, which may contribute to the accumulation of autophagosomes. Moreover, inhibition of RIP3 by GSK’872, a RIP3-selective kinase inhibitor, attenuated the accumulation of both LC3II and p62 (Fig. 1C, D), indicating that the inhibition of RIP3 restored the autophagic degradation.

**Fig. 2:**
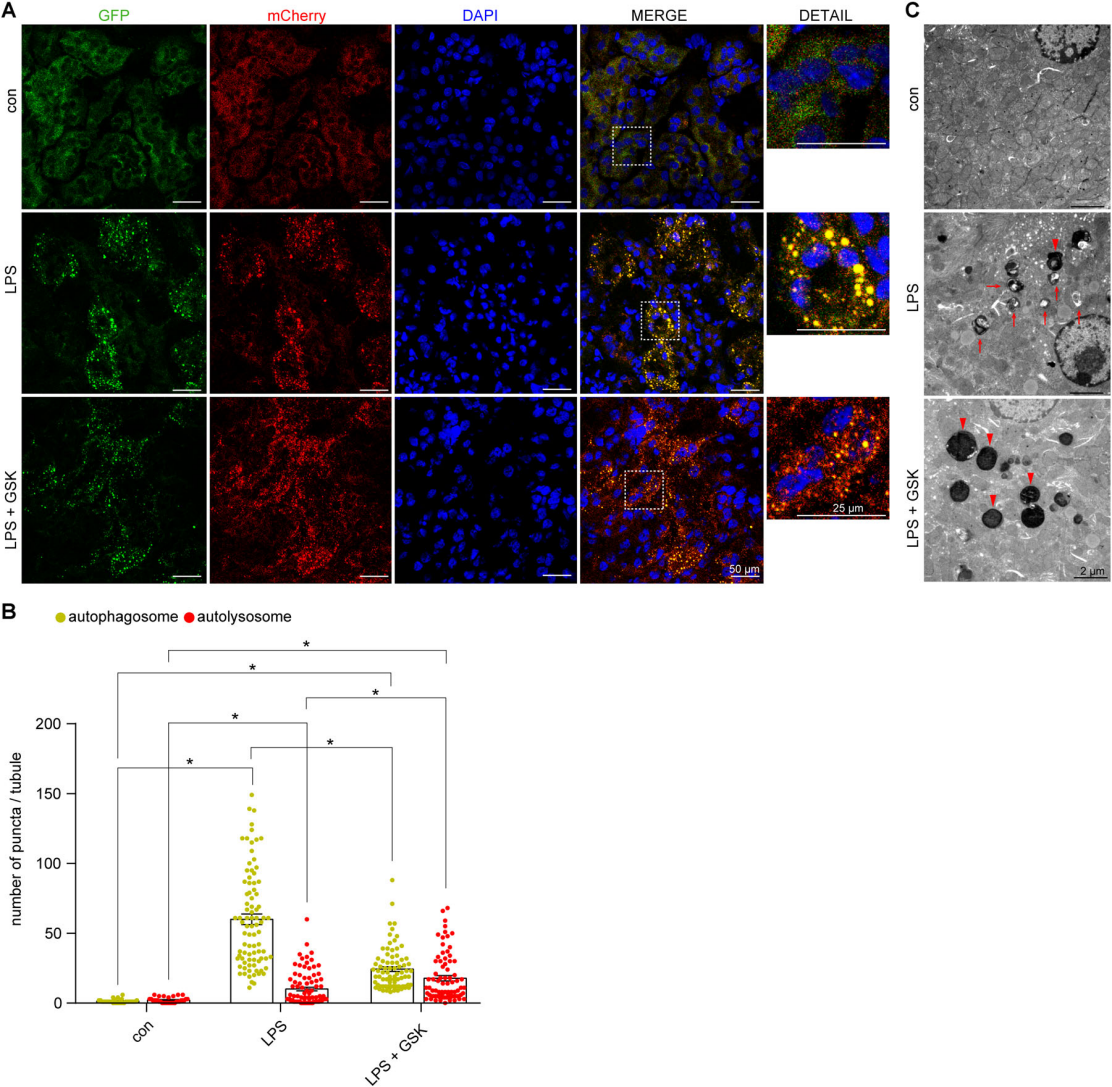
RIP3 was activated to suppress autophagic flux in septic AKI mice. **A** Confocal laser scanning microscopy images of kidney tissues from renal tubular cell-specific mCherry-GFP-LC3 reporter mice treated as indicated. Scale bar = 50 μm for the left four lanes, 25 μm for the detailed images. **B** Quantitative analysis showed few autophagosomes (yellow puncta) and autolysosomes (red-only puncta) were detected in the renal tubular cells of the control group. The number of autophagosomes were largely increased, with a slight increase in the number of autolysosomes, in the LPS group. GSK induced the formation of autolysosomes and decreased the LPS-induced accumulation of autophagosomes (*n* = 3–4, 32–86 tubules from each group). **C** TEM showed that few autophagic vacuoles were detected in the renal tubular cells of the control mice. A lot of initial autophagic vacuoles (arrows) but few late autophagic vehicles (arrowheads) were detected in the renal tubular cells of LPS-induced mice. GSK induced the formation of late autophagic vehicles and decreased the number of initial autophagic vacuoles. Scale bar = 2 μm. *P < 0.05.

To explore the role of RIP3 in autophagic flux, the renal tubular cell-specific mCherry-GFP-LC3 autophagy reporter mice were used (Fig. 2A, B). Under LPS treatment, the number of autophagosomes (yellow puncta) was largely increased, whereas there was a slight increase in the number of autolysosomes (red-only puncta) compared to the control group. Inhibition of RIP3 upregulated the number of autolysosomes and downregulated the number of autophagosomes (Fig. 2B). Similarly, results from TEM showed an accumulation of initial autophagic vacuoles without obvious formation of late autophagic vehicles in the renal tubular cells of the septic AKI mice compared to the control group. Inhibition of RIP3 led to an increase of the formation of late autophagic vehicles and a decrease of the initial autophagic vacuoles (Fig. 2C). Thus, these results suggested that RIP3 inhibited the formation of autolysosomes, inhibiting the clearance of autophagosomes during septic AKI.

In accordance of the restoration of autophagic degradation, inhibition of RIP3 remarkably attenuated pathological changes of renal tubules, as well as the levels of serum creatinine and BUN as we reported previously (Supplementary Fig. 2a–c)^9^. Taken together, these results showed that RIP3 was activated to suppress autophagic degradation and lead to tubular injury and renal dysfunction in septic AKI.

### RIP3 was activated to suppress autophagic degradation in LPS-induced cultured PTECs in vitro

**Fig. 3:**
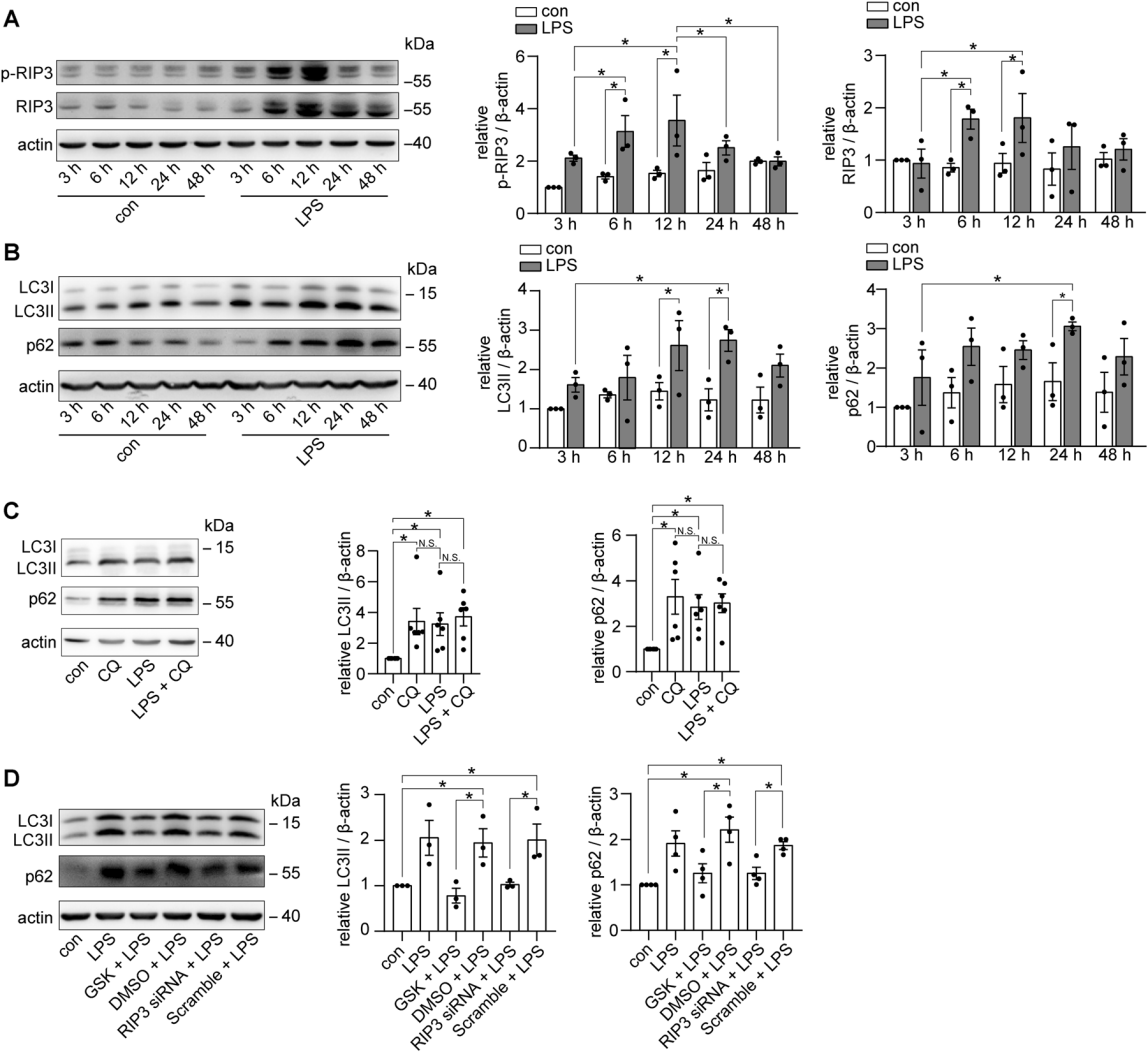
RIP3 activation inhibited autophagic degradation in LPS-induced cultured PTECs. **A**, **B** Cultured PTECs were treated with DMSO or LPS for different time points as indicated. **A** Immunoblotting showed that the expression of p-RIP3 and RIP3 was increased at 6 and 12 h LPS treatment (*n* = 3). **B** Immunoblotting showed an increase in the expression of LC3II and p62 at 24 h LPS treatment (*n* = 3). **C** Cultured PTECs were treated with the lysosome inhibitor chloroquine (CQ), LPS, or LPS plus CQ for 24 h. Immunoblotting showed that the addition of CQ did not further increase the levels of LC3II and p62 in LPS-treated PTECs (*n* = 6). **D** Immunoblot of LC3 and p62 in cultured PTECs treated with LPS, LPS plus GSK’872 (GSK), LPS plus DMSO, LPS plus RIP3 siRNA, or LPS plus scrambled siRNA (scramble) for 24 h. GSK or RIP3 siRNA downregulated the LPS-induced increase of LC3II and p62 (*n* = 3–4). *P < 0.05. NS for nonsignificant.

**Fig. 4:**
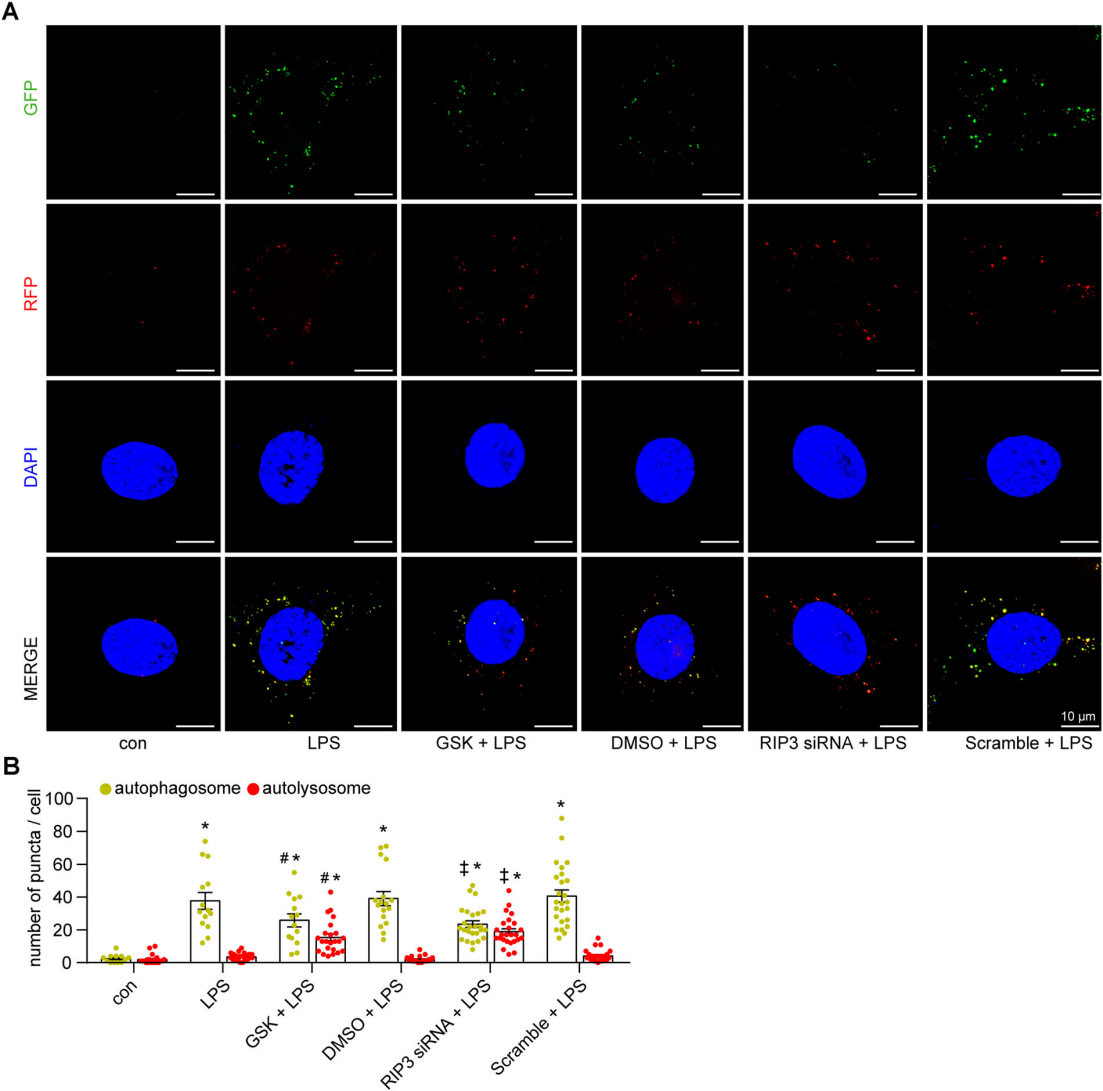
RIP3 activation suppressed autophagic flux in LPS-induced cultured PTECs. **A**, **B** Cultured PTECs transfected with mRFP-GFP-LC3 adenovirus and treated as indicated for 24 h. Representative confocal laser scanning microscopy images (**A**) and quantitative analysis (**B**) of autophagosomes (yellow puncta) and autolysosomes (red-only puncta). Few autophagosomes and autolysosomes were observed in the control group. The number of autophagosomes but not autolysosomes was increased in the LPS-treated PTECs (LPS, LPS + DMSO, and LPS + Scramble group). GSK or RIP3 siRNA induced the formation of autolysosomes, whereas it decreased the accumulation of autophagosomes in LPS-induced PTECs (*n* = 3–4, 21–26 cells from each group). *P < 0.05, significantly different from the control group. ^#^*P* < 0.05, significantly different from the DMSO + LPS group. ^‡^*P* < 0.05, significantly different from the scramble + LPS group.

Consistent with the results of our in vivo study, the activity of RIP3 was increased in a time-dependent manner in LPS-induced cultured PTECs (Fig. 3A). The level of LC3II was increased with a peak at 24 h of LPS treatment, when p62 was also markedly increased (Fig. 3B). Moreover, the lysosome inhibitor CQ^30^ did not increase the levels of LCII or p62 in LPS-induced cultured PTECs (Fig. 3C), further supporting that the LPS-induced accumulation of LC3II and p62 was caused by impaired autophagic degradation. The LPS-induced accumulation of LC3II and p62 was attenuated by inhibiting RIP3 pharmacologically by GSK’872 or genetically by RIP3 siRNA (Fig. 3D and Supplementary Fig. 3a). Also, in the cultured PTECs transfected with the mRFP-GFP-LC3 adenovirus, many autophagosomes (yellow puncta) appeared without obvious autolysosomes (red-only puncta) formation at 24 h of LPS treatment compared to control group. Inhibition of RIP3 induced the formation of autolysosomes and decreased accumulated autophagosomes under LPS treatment (Fig. 4A, B). These results further confirmed the insufficiency of autophagic degradation mediated by RIP3 during septic AKI.

### RIP3 inhibited the nuclear translocation of TFEB and interacted with TFEB during septic AKI

**Fig. 5:**
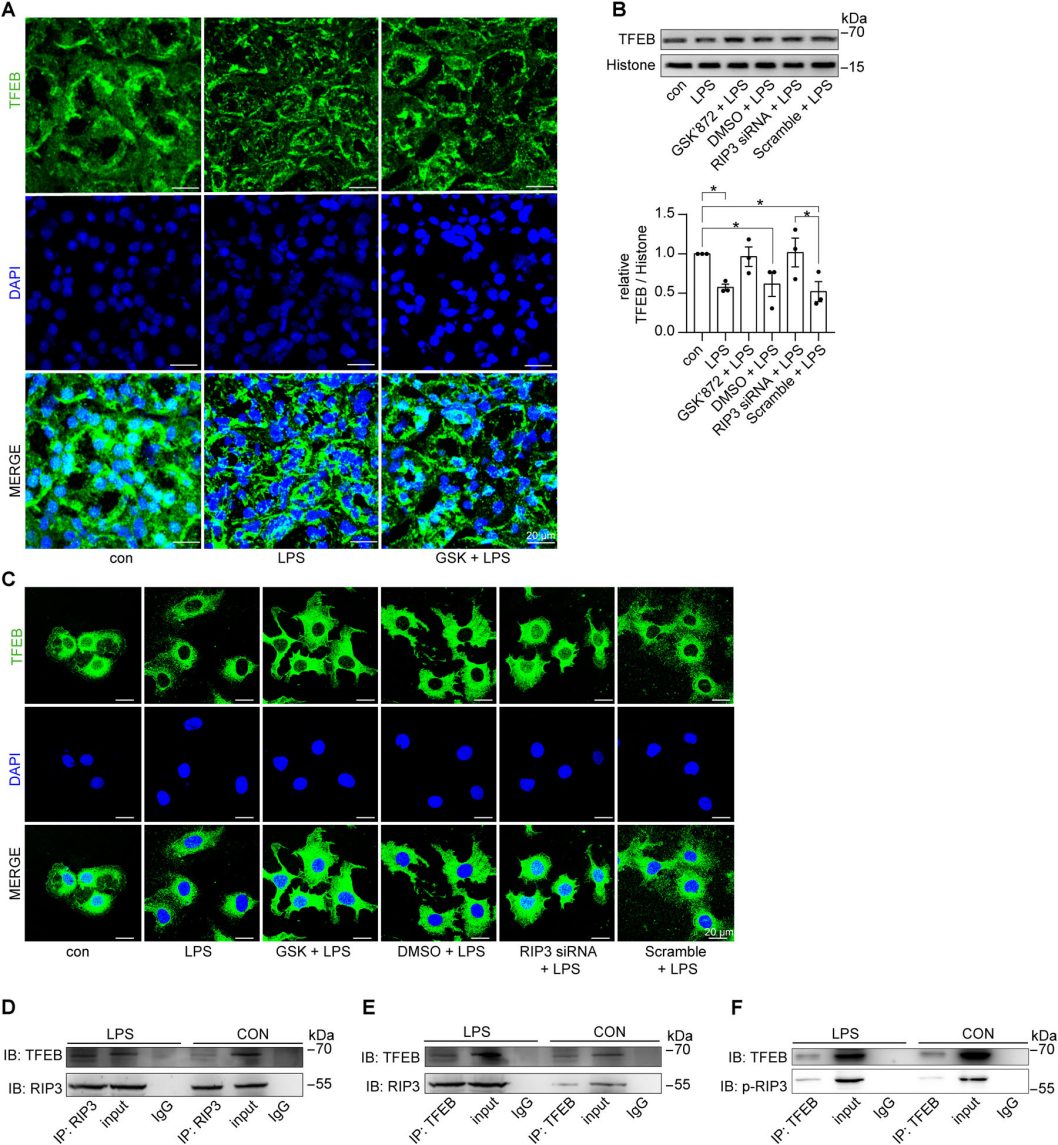
RIP3 impeded the nuclear translocation of TFEB in septic AKI mice and LPS-treated cultured PTECs. **A** Immunofluorescence staining for TFEB (green) and DAPI (blue) in the kidneys from C57BL/6 mice treated with sterilized saline (con), LPS, or LPS plus GSK’872 (GSK) for 24 h. GSK partially restored the decreased nuclear staining of TFEB in the renal tubular cells of LPS-induced mice. Scale bar = 20 μm. **B** Cultured PTECs treated with LPS, LPS plus GSK, LPS plus DMSO, LPS plus RIP3 siRNA, or LPS plus scrambled siRNA (Scramble) for 12 h. The nuclear protein fractions were immunoblotted for TFEB. Histone was used as the nuclear marker. TFEB expression against Histone was decreased by LPS treatment, which was restored by GSK or RIP3 siRNA (*n* = 3). **C** Immunofluorescence staining for TFEB (green) and DAPI (blue) in cultured PTECs treated as indicated. The nuclear translocation of TFEB was inhibited by LPS, which was rescued by GSK or RIP3 siRNA. Scale bar = 20 μm. **D**, **E** Lysates from cultured PTECs treated with LPS or DMSO (con) for 12 h were subjected to immunoprecipitation using an anti-RIP3 antibody (**D**) or anti-TFEB antibody (**E**), and IgG antibody followed by immunoblot for TFEB and RIP3. Input proteins were detected with anti-RIP3 and anti-TFEB antibodies. RIP3 interacted with TFEB in LPS-treated PTECs compared to the control group. **F** Lysates from cultured PTECs treated with LPS or DMSO (con) for 12 h were subjected to immunoprecipitation using anti-TFEB antibody and IgG antibody followed by immunoblot for p-RIP3. Input proteins were detected with anti-p-RIP3 and anti-TFEB antibodies. p-RIP3 interacted with TFEB in LPS-treated PTECs compared to the control group. *P < 0.05.

The lysosome serves as the degradation hub for autophagy, which is first fused with autophagosomes and then degraded the autophagosomal cargo and the autophagosome itself. We then examined whether TFEB, a master regulator of both the lysosome and autophagy pathways, was involved in RIP3-mediated autophagic degradation suppression in septic AKI. First, the nuclear translocation of TFEB was inhibited in the renal tubular cells of septic AKI mice, which was partially restored by inhibition of RIP3 (Fig. 5A). Also, the decreased nuclear translocation of TFEB was confirmed in LPS-treated cultured PTECs, which was rescued by suppressing RIP3 through GSK’872 or RIP3 siRNA (Fig. 5B, C). Moreover, results of CoIP assay showed an interaction between RIP3 and TFEB, as well as an interaction between p-RIP3 and TFEB in LPS-induced cultured PTECs compared to the control group (Fig. 5D–F). These results suggested that RIP3 was activated to interact with TFEB and inhibit the nuclear translocation of TFEB. In addtion, the nuclear translocation of TFEB showed marginal change by inhibiting MLKL, the mediator of RIP3-induced canonical necroptotic signaling, under LPS treatment (Supplementary Fig. 4a–c), suggesting that the inhibition of TFEB was independent of MLKL in septic AKI. This was further supported by the marginal change of the accumulated LC3II level by inhibiting MLKL under LPS treatment (Supplementary Fig. 4d).

### RIP3 resulted in lysosome dysfunction during septic AKI

**Fig. 6:**
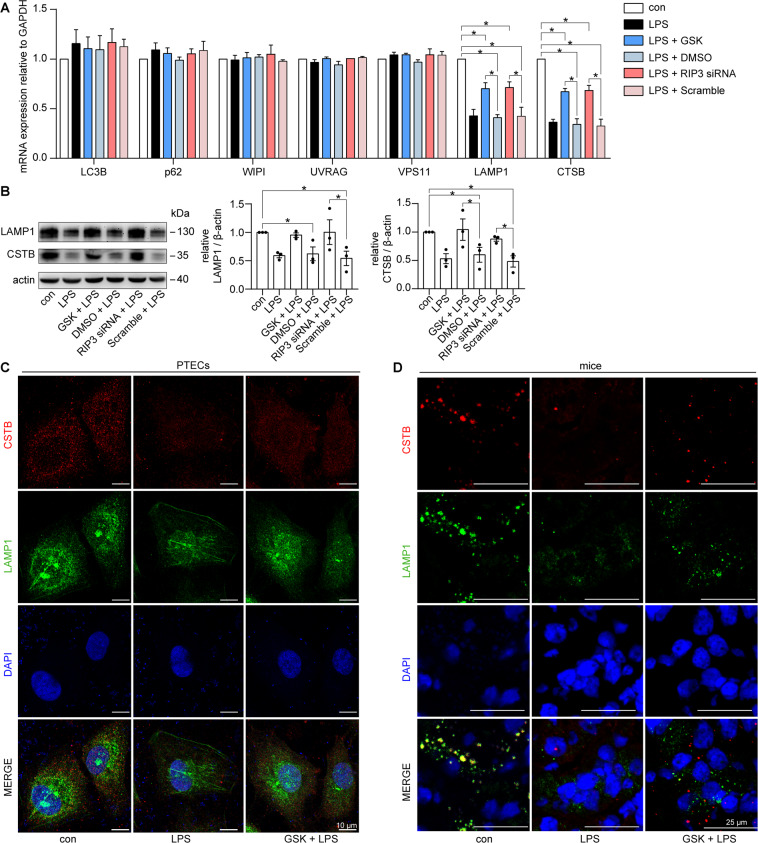
The expression of LAMP1 and CTSB was suppressed by RIP3 in LPS-induced cultured PTECs and septic AKI mice. **A** RT-qPCR analyses of the expression of potential TFEB target genes in cultured PTECs treated with LPS, LPS plus GSK’872 (GSK), LPS plus DMSO, LPS plus RIP3 siRNA, or LPS plus scrambled siRNA (Scramble) for 12 h. The mRNA levels of LAMP1 and CTSB were inhibited by LPS, which was restored by GSK or RIP3 siRNA (*n* = 3). **B** Immunoblotting showed that the protein levels of LAMP1 and CTSB was decreased by LPS, which was restored by GSK’872 or RIP3 siRNA in cultured PTECs treated as indicated for 24 h (*n* = 3). **C** Immunofluorescence staining for CTSB (red), LAMP1 (green), and DAPI (blue) in cultured PTECs treated as indicated for 24 h. GSK restored the decreased expression of CTSB and LAMP1 in LPS-treated PTECs. Scale bar = 10 μm. **D** Immunofluorescence staining for CTSB (red), LAMP1 (green), and DAPI (blue) in the kidneys from C57BL/6 mice treated with vehicle (con), LPS, or LPS plus GSK for 24 h. The expression of CTSB and LAMP1 were suppressed in the renal tubular cells of LPS-induced mice, which were partially restored by GSK. Scale bar = 25 μm. *P < 0.05.

TFEB directly promotes the transcription of several lysosome-related and autophagy-related genes^22,31^. We explored the effect of RIP3 on the activity of TFEB during septic AKI by testing the mRNA levels of several reported potential TFEB targets genes^31^. Among them, the mRNA levels of LAMP1 (marker for lysosome biogenesis^32,33^) and CTSB (responsible for driving proteolytic degradation within the lysosome^34^) were significantly downregulated in LPS-induced cultured PTECs compared to the control group, which was rescued by inhibition of RIP3 through GSK’872 or RIP3 siRNA (Fig. 6A). Moreover, the inhibition of RIP3 restored the decreased protein levels of LAMP1 and CTSB in LPS-induced PTECs (Fig. 6B, C) and in the renal tubular cells of LPS-induced mice (Fig. 6D). Taken together, these results suggested that RIP3 led to lysosome dysfunction, including impairment of lysosome biogenesis and degradation, during septic AKI.

### Inhibition of TFEB-mediated autophagic degradation suppression in LPS-induced cultured PTECs

**Fig. 7:**
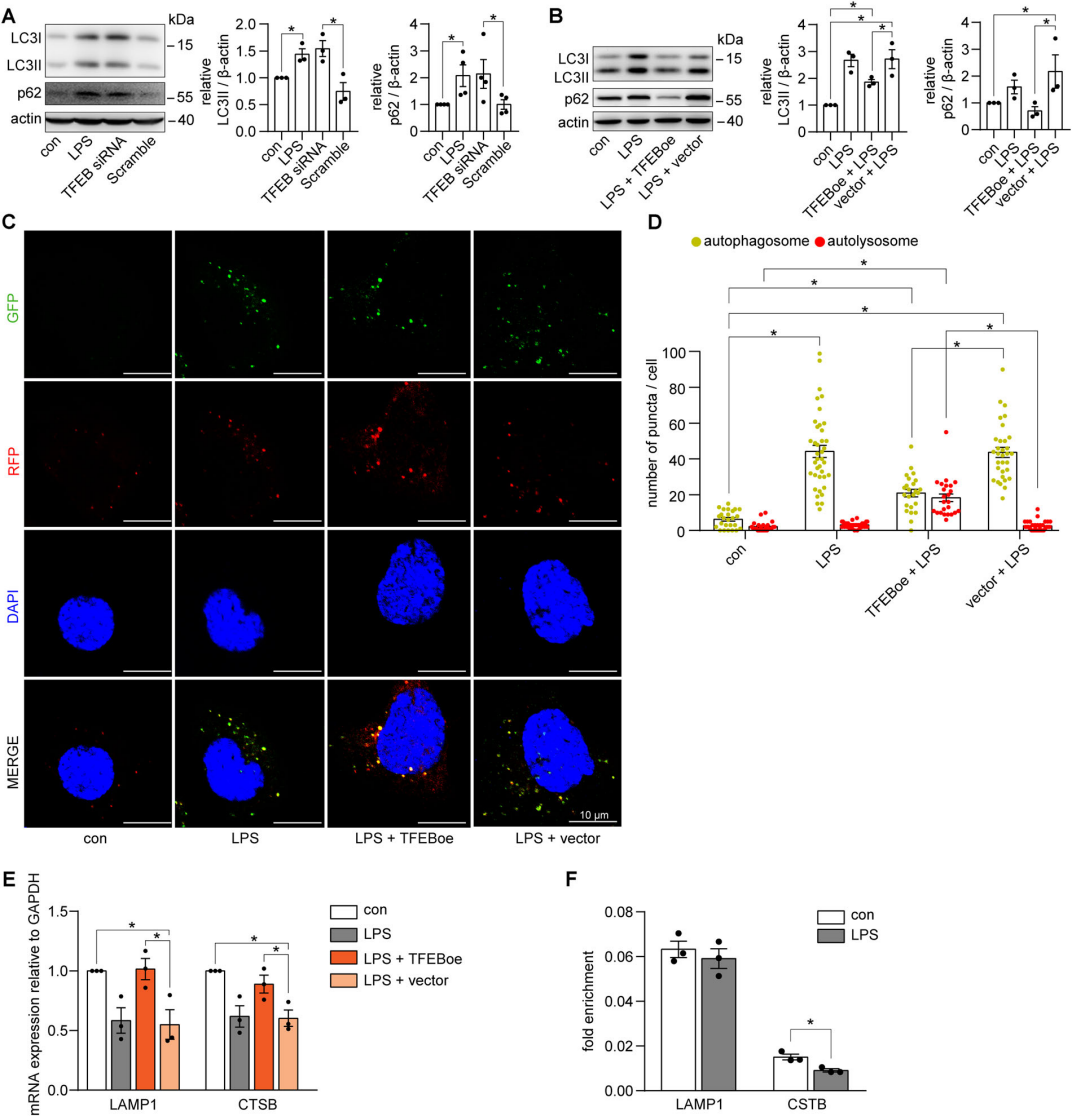
TFEB regulated the autophagic degradation in LPS-treated cultured PTECs. **A** Immunoblot of LC3 and p62 in cultured PTECs treated with LPS, TFEB siRNA, or Scramble siRNA (scramble) for 24 h. Similar to LPS treatment, TFEB siRNA led to an accumulation of LC3II and p62 (*n* = 3–4). **B** Immunoblotting of LC3 and p62 in cultured PTECs treated with LPS, LPS plus TFEB-overexpression adenovirus (TFEBoe), or LPS plus vector adenovirus (vector) for 24 h. Overexpression of TFEB decreased the accumulation of LC3II and p62 in LPS-treated PTECs (*n* = 3). **C**, **D** Cultured PTECs transfected with mRFP-GFP-LC3 adenovirus and treated as indicated for 24 h. Representative confocal laser scanning microscopy images (**C**) and quantitative analysis (**D**) of autophagosomes (yellow puncta) and autolysosomes (red-only puncta). Overexpression of TFEB induced the formation of autolysosomes, whereas it decreased the accumulation of autophagosomes in LPS-induced PTECs (*n* = 3–4, 25–39 cells from each group). Scale bar = 10 μm. *P < 0.05. **E** RT-qPCR analyses of the expression of LAMP1 and CTSB in cultured PTECs treated as indicated for 12 h. Overexpression of TFEB rescued the decreased mRNA levels of LAMP1 and CTSB in LPS-treated PTECs (*n* = 3). **F** ChIP analyses of the binding of TFEB to the promoters of *LAMP1* and *CTSB* in cultured PTECs treated as indicated for 24 h, using an antibody to TFEB. IgG was used as a negative control. Quantitative PCR was conducted to measure the immunoprecipitated DNA using a promoter-specific primer. TFEB binding to the promoter of *CTSB* but not *LAMP1* was inhibited in the LPS-treated cultured PTECs (*n* = 3). Data were expressed as faction of input. *P < 0.05.

We explored the role of TFEB in LPS-induced lysosome-autophagy disorder in cultured PTECs. First, similar to LPS treatment, TFEB siRNA induced an accumulation of both LC3II and p62 (Fig. 7A and Supplementary Fig. 3b). Next, overexpression of TFEB alleviated the LPS-induced accumulation of LC3II and p62 (Fig. 7B and Supplementary Fig. 3c) and restored the autolysosome formation (Fig. 7C, D). Moreover, overexpression of TFEB restored the decreased mRNA levels of LAMP1 and CTSB (Fig. 7E). We further utilized ChIP assay to find that LPS impeded TFEB binding to the promoters of *CTSB* but not *LAMP1* (Fig. 7F). Collectively, these results suggested that TFEB was critical for the maintenance of autophagy in renal tubular cells under physiological conditions and inhibition of TFEB mediated the autophagic degradation suppression in LPS-treated cultured PTECs.

### RIP3-TFEB pathway was involved in the patient with septic AKI

**Fig. 8:**
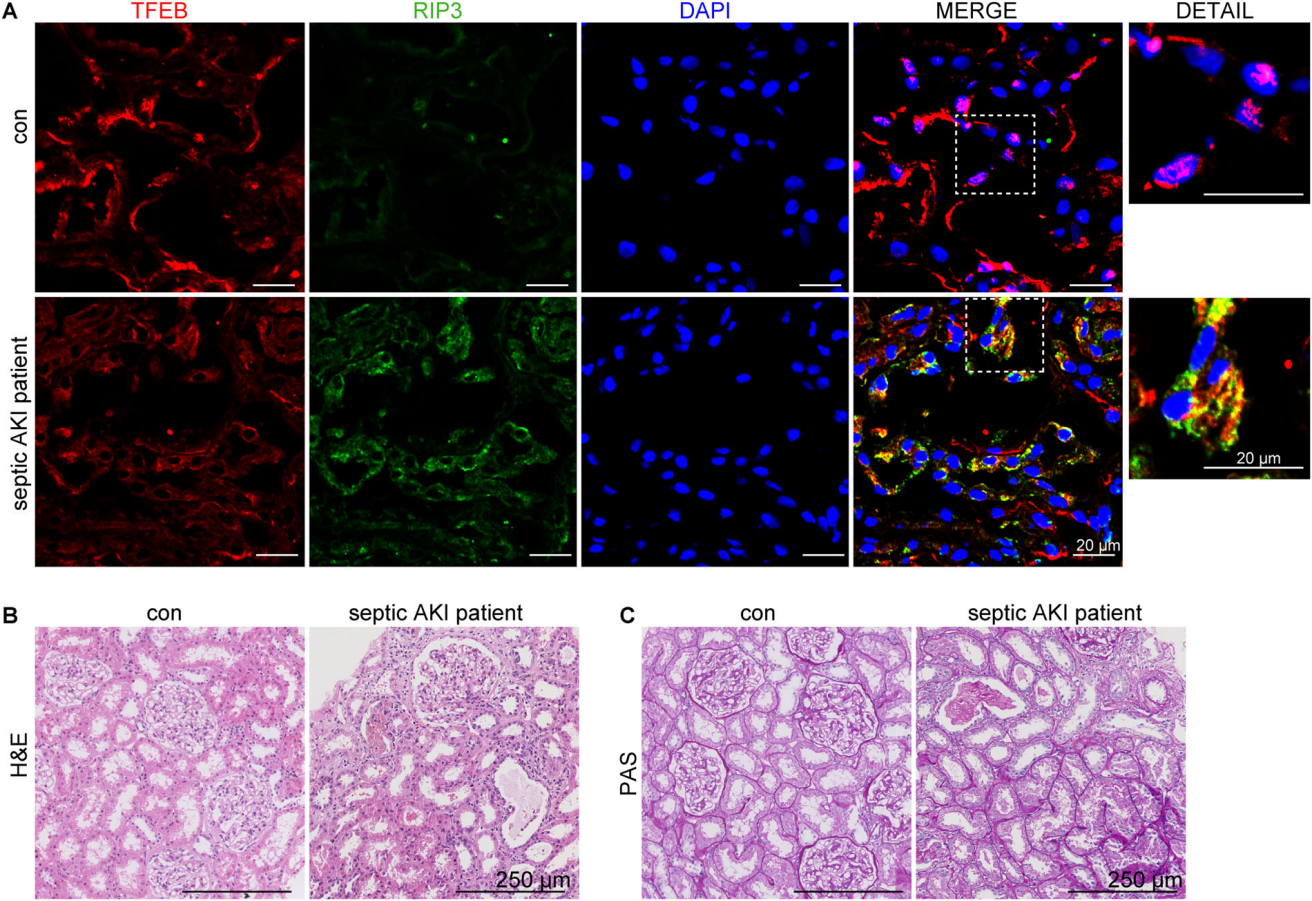
RIP3-TFEB pathway was involved in the patient with septic AKI. **A** Immunofluorescence staining for TFEB (red), RIP3 (green), and DAPI (blue) of kidney specimens from one control subject and one patient with septic AKI. The RIP3 expression was enhanced, whereas the TFEB nuclear translocation was inhibited in the renal tubular cells in the septic AKI patient. TFEB colocalized with RIP3 in the cytosol in the renal tubular cells in the septic AKI patient. Scale bar = 20 μm. **B**, **C** Representative histology images of hematoxylin–eosin (H&E) staining and periodic acid–Schiff (PAS) staining of renal tissues from the septic AKI patient and the control subject. Scale bar = 250 μm.

We examined the expression of RIP3 and TFEB in kidney tissues from the septic AKI patient. Consistent with our in vivo and in vitro results, we observed an enhancement of RIP3 expression and an inhibition of TFEB nuclear translocation in the renal tubular cells in the septic AKI patient, accompanied by severe tubular injury (Fig. 8A–C). Notably, some parts of the cytosolic TFEB colocalized with RIP3 in the cytosol of the renal tubular cells from the septic AKI patient but not in the control subject (Fig. 8A), further supporting an interaction of RIP3 and TFEB during septic AKI.

## Discussion

RIP3 has recently been identified to play a pivotal role in tubular injury and renal dysfunction in septic AKI^8^. Our recent study showed that RIP3 inhibitor protected against LPS-induced renal tubular cell death and kidney damage^9^. However, it remained elusive as to how RIP3 mediated septic AKI and how the RIP3 inhibitor protected renal tubules and the kidney. The present study has demonstrated that RIP3 was activated to suppress autophagic degradation in septic AKI mice and in LPS-treated cultured PTECs. Mechanistically, this study has, for the first time to our knowledge, demonstrated that RIP3 inhibited the nuclear translocation and activity of TFEB, further leading to lysosome dysfunction and autophagic degradation suppression. In addition, the results suggested an interaction between RIP3 and TFEB, as well as p-RIP3 and TFEB under septic conditions, which may be related to the inhibition of TFEB by RIP3. Also, our study suggested that the RIP3 inhibitor may protect the kidney by restoring the autophagic degradation during septic AKI. Thus, the present study has delineated a pivotal role of the RIP3-TFEB pathway implicated in the autophagic degradation suppression during septic AKI, providing potential therapeutic targets for the prevention and treatment of septic AKI.

RIP3, a major regulator of the necroptosis pathway, has been implicated in ischemia-reperfusion- and cisplatin-induced AKI^35–39^. Recently, the pathological role of RIP3 in contributing to renal injury during septic AKI has been reported by Sureshbabu et al.^8^ (genetically deleting RIP3) and our previous study (inhibiting RIP3 using a RIP3-selevtive inhibitor)^9^ in septic AKI mice and cultured renal tubular cells. Our present study has demonstrated the regulation of TFEB-autophagy pathway by RIP3, providing new insights into the involvement of RIP3 in AKI. Interestingly, we observed that the inhibition of TFEB-autophagy pathway was independent of MLKL in septic AKI, supporting a necroptosis-independent pathogenetic role of RIP3 in septic AKI reported by Sureshbabu et al.^8^.

In this study, we have identified RIP3 as a negative regulator of autophagy during septic AKI. First, RIP3 was activated in renal tubular cells of the patient with septic AKI, kidneys of LPS-induced mice, and LPS-induced cultured PTECs. The activation of RIP3 was accompanied by an accumulation of the autophagosome marker LC3II and the autophagic substrate p62 under LPS treatment in vivo and in vitro. The lysosome inhibitor did not further increase the levels of LCII or p62 in LPS-treated PTECs, suggesting that the LPS-induced accumulation of LC3II and p62 was caused by impaired autophagic degradation. Second, inhibition of RIP3 attenuated the LPS-induced accumulation of p62 in vivo and in vitro, suggesting RIP3 suppressed the clearance of autophagic substrate. Also, the LPS-induced accumulation of LC3II was attenuated by inhibition of RIP3, which may be a result of the restoration of autophagic degradation to eliminate the autophagosomes. To verify this, we explored the role of RIP3 in autophagic flux by using renal tubular cell-specific mCherry-GFP-LC3 autophagy reporter mice in vivo and PTECs overexpressing mRFP-GFP-LC3 in vitro. Inhibition of RIP3 induced autolysosome formation and decreased the accumulated autophagosomes under LPS treatment. Moreover, inhibition of RIP3 rescued the LPS-induced downregulation of LAMP1 and CTSB in vivo and in vitro, suggesting RIP3 resulted in lysosome dysfunction during septic AKI. Taken together, these results have demonstrated that RIP3 suppressed autophagic degradation during septic AKI.

The effect of RIP3 on autophagy has been reported inconsistently in other cells or tissues. RIP3 was shown to induce the conversion of LC3I to LC3II in osteosarcoma cells^40^ and glioblastoma cells^41^. In contrast with the present study, RIP3 positively regulated autophagy in intestinal epithelial cells^42^. In view of the discrepancy between these studies and ours, it is implicated that the effect of RIP3 on autophagy may vary from different types of cells and tissues. It is noteworthy that the autophagic flux is a dynamic process with multi-steps. There is a significant lack of understanding of which steps of the autophagic flux was targeted by RIP3. Our results suggested that RIP3 targeted the late stage of the autophagic flux at least partially by regulating autolysosome formation, lysosome biogenesis, and degradation.

The present study provides novel insights into the molecular mechanisms underlying the role of RIP3 in autophagic degradation. The nuclear translocation of TFEB was inhibited in the renal tubular cells of the patient with septic AKI, septic AKI mice, as well as the LPS-induced cultured PTECs. Also, the activity of TFEB was decreased during septic AKI as evidenced by the downregulation of both the mRNA and protein levels of CTSB, a reported potential target gene of TFEB^31^. ChIP assay was further utilized to confirm the decrease of TFEB activity. Importantly, inhibition of RIP3 restored this decreased nuclear translocation and activity of TFEB in vitro and in vivo. Collectively, these results have identified RIP3 as a novel regulator of TFEB, which suppressed the nuclear translocation and activity of TFEB during septic AKI.

We next explored how RIP3 regulated TFEB. In this study, we showed that TFEB colocalized with RIP3 in the cytosol of the renal tubular cells of the patient with septic AKI. We further used CoIP assay to confirm an interaction between RIP3 and TFEB under septic conditions. Moreover, we observed an interaction between p-RIP3 and TFEB, suggesting that this interaction between RIP3 and TFEB was related to the phosphorylation of RIP3. Activated RIP3 interacted with TFEB and suppressed the nuclear translocation of TFEB. Previous study has demonstrated that the phosphorylation of RIP3 is required for its function involving in phosphorylating its target proteins^43^. It has also been reported that the subcellular location and activity of TFEB could be regulated through the phosphorylation or dephosphorylation of TFEB by several other serine threonine kinases including mTOR, ERK, and Calcineurin^44^. Further study needs to elucidate whether activated RIP3 regulate TFEB via direct phosphorylation.

Recently, TFEB-related lysosome and autophagy disorder has been implicated in several diseases including liver steatosis and neurodegenerative storage diseases^45–48^. In 2018, Lynch et al.^49^ showed that the protein levels of TFEB was decreased in cisplatin-treated cultured renal collecting duct cells and inhibition of TFEB exacerbated cell death rate in these cells. Although they did not delineate the role of TFEB in renal tubular cells and in septic AKI, their study indicated that TFEB may have a renal-protective role. The present study showed that in normal cultured PTECs, TFEB siRNA led to the accumulation of LC3II and p62. In LPS-treated cultured PTECs and the renal tubular cells of LPS-induced mice, TFEB was inhibited. Overexpression of TFEB alleviated the aberrant accumulation of LC3II and p62, and restored the autolysosome formation, CTSB, and LAMP1 in LPS-treated cultured PTECs. Thus, these results provided evidence that TFEB may be essential for maintaining the autophagy in renal tubular cells under physiological conditions and inhibition of TFEB mediated the autophagic degradation suppression during septic AKI. Notably, the binding of TFEB to the promoter of *CTSB* but not *LAMP1* was suppressed in LPS-treated cultured PTECs measured by ChIP assay, suggesting that TFEB regulated CTSB at a transcription level, while having a transcription-independent effect on LAMP1 in renal tubular cells during septic AKI.

GSK’872, a selective inhibitor of RIP3, binds to the kinase domain of RIP3 to inhibit its kinase activity^50,51^. Notably, Mandal et al.^51,52^ found that a high dose of GSK’872 could result in a conformational change of RIP3, which promoted the cell apoptosis pathway independent of its kinase activity. Our study also has provided a reference for the safe and effective concentration of GSK’872 for the regulation of autophagy in AKI mice models.

An obvious weakness of our study is that only one septic AKI patient was examined. It is generally understood that the patients with sepsis and sepsis-induced AKI are often accompanied by multiple organ dysfunction and unstable hemodynamics, making it highly risky and often unethical to do biopsy. For this reason, very few studies collected renal biopsy from septic AKI patients and, if they did, only very few patients were examined. With the help of our intensive care unit, we worked very hard to obtain renal biopsies of one sepsis AKI patient. Also, it is important to note that our study is mainly based on cell and animal models, whereas the human biopsy examination is only to implicate the clinical relevance of our preclinical data.

In conclusion, the present study has identified a pivotal role of RIP3 in suppressing autophagic degradation during septic AKI. We have unveiled RIP3 as a novel regulator of TFEB by interacting with and inhibiting the nuclear translocation and activity of TFEB, leading to lysosome dysfunction and autophagic degradation suppression in septic AKI. The results also suggested the RIP3 inhibitor may protect the kidney by restoring the autophagic degradation in renal tubular cells during septic AKI.

## Supplementary information

Supplementary Information

Supplementary figure legends

Supplementary Figure1

Supplementary Figure 2

Supplementary Figure 3

Supplementary Figure 4

Supplementary Table 1

Supplementary Table 2

Supplementary Table 3

Supplementary Table 4

## Data Availability

The datasets used and/or analyzed during the current study are available from the corresponding author on reasonable request.
