# Supplementary material for: RIP3 impedes transcription factor EB to suppress autophagic degradation in septic acute kidney injury
Source: Cell Death Dis. 2021 Jun 8;12(6):593. doi: 10.1038/s41419-021-03865-8 (PMC8187512; doi:10.1038/s41419-021-03865-8)
Supplement: Supplementary file 1 — Supplementary Information [file 41419_2021_3865_MOESM1_ESM.docx]

**Supplementary Information**

Supplementary figure legends

Supplementary figure 1

Supplementary figure 2

Supplementary figure 3

Supplementary figure 4

Supplementary Table 1

Supplementary Table 2

Supplementary Table 3

Supplementary Table 4
