# Supplementary material for: RIP3 impedes transcription factor EB to suppress autophagic degradation in septic acute kidney injury
Source: Cell Death Dis. 2021 Jun 8;12(6):593. doi: 10.1038/s41419-021-03865-8 (PMC8187512; doi:10.1038/s41419-021-03865-8)
Supplement: Supplementary file 2 — Supplementary figure legends [file 41419_2021_3865_MOESM2_ESM.docx]

**Supplementary figure legends**

**Supplementary Fig. 1 Generation of the renal tubular cells-specific mCherry-GFP-LC3 autophagy reporter mouse model.** (I) The targeting vector containing the following components were constructed: CMV enhancer/chicken beta-actin core promoter(CAG)-loxP-Stop sequence-loxP-mCherry-linker-GFP-linker-LC3-WPRE-bGH-poly(A)-Frt-PGK-Neo-bGH poly(A)-Frt. Targeting of the transgene cassettes into intron 1 of the Rosa26 locus was accomplished via standard homologous recombination in JM8A3 embryonic stem (ES) cells. Correctly targeted ES cells were injected into blastocysts to obtain the chimeric mice. (II) The resulting chimeric mice were crossed to FLP-deleter mice to remove the Neo cassette. (III) R26-LSL-mCherry-GFP-LC3 heterozygous mice were crossed to Ksp-Cre transgenic mice. R26-LSL-mCherry-GFP-LC3/R26-LSL-mCherry-GFP-LC3; Ksp-Cre mice were used as experimental group in this study.

**Supplementary Fig. 2 RIP3 led to renal tubular cell injury and renal dysfunction in septic AKI mice. a** Hematoxylin-eosin (H&E) staining of mice kidney tissues showed the LPS-induced tubular vacuolization, swelling and loss of brush border was restored by GSK’872 (GSK) at 12 h- and 24 h-LPS treatment. Scale bar = 50 μm. **b** Semiquantitative analysis of tubular injury from 10 random fields (n=3). **c** Blood samples were collected for measuring serum creatinine (SCr) and BUN. GSK significantly attenuated the LPS-induced increase of SCr and BUN at 24 h-LPS treatment (n=6). ^*^*P* < 0.05, significantly different from the control group. ^#^*P* < 0.05, significantly different from the LPS group.

**Supplementary Fig. 3 Efficiency of RIP3 or TFEB interference in cultured renal proximal tubular epithelial cells (PTECs). a** Cultured PTECs were transfected with RIP3 siRNA or scrambled siRNA (Scramble). The total protein extractions were subjected to immunoblotted for TFEB and β-actin. **b** Cultured PTECs were transfected with TFEB siRNA or scrambled siRNA (Scramble) (n=3). The nuclear protein fractions were immunoblotted for TFEB. Histone was used as the nuclear marker. **c** Cultured PTECs were transfected with TFEB overexpression adenovirus (TFEBoe) or empty-vector (vector). The nuclear protein fractions were immunoblotted for TFEB. Histone was used as the nuclear marker. (n=3). ^*^*P* < 0.05.

**Supplementary Fig. 4 MLKL inhibition didn’t affect the nuclear translocation of TFEB and the level of autophagosomes under LPS treatment. a** Cultured PTECs were transfected with three MLKL siRNAs with different sequences or scrambled siRNA (Scramble). The total protein extractions were subjected to immunoblotted for MLKL and β-actin. The MLKL siRNA-3 significantly decreased the protein expression of MLKL (n=3), which was used in the subsequent experiments (named “MLKL siRNA”). **b** Cultured PTECs treated with LPS, LPS plus scrambled siRNA (Scramble), LPS plus MLKL siRNA for 24 h. The nuclear protein fractions were immunoblotted for TFEB. Histone was used as the nuclear marker. TFEB expression against Histone was decreased by LPS treatment, which was not affected by MLKL siRNA (n=4). **c** Immunofluorescence staining for TFEB (red) and DAPI (blue) in cultured PTECs treated as indicated. The nuclear translocation of TFEB was inhibited by LPS, which was not affected by MLKL siRNA. Scale bar = 20 μm. **d** Cultured PTECs treated with LPS, LPS plus scrambled siRNA (Scramble), LPS plus MLKL siRNA for 24 h. The total protein extractions were subjected to immunoblotted for LC3 and β-actin. The LPS-induced accumulation of LC3II was not affect by MLKL siRNA (n=3). ^*^*P* < 0.05. N.S. for non-significant.
