# Supplementary figures and images for: RIP3 impedes transcription factor EB to suppress autophagic degradation in septic acute kidney injury
Source: Cell Death Dis. 2021 Jun 8;12(6):593. doi: 10.1038/s41419-021-03865-8 (PMC8187512; doi:10.1038/s41419-021-03865-8)

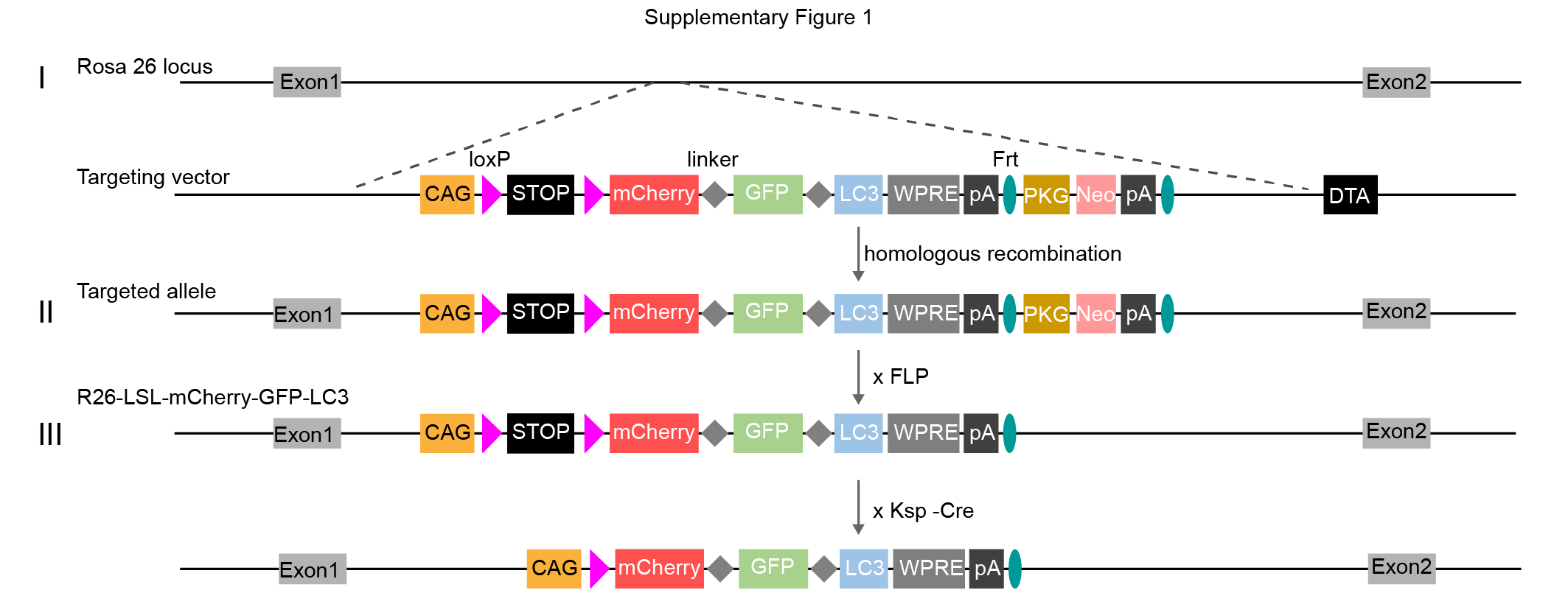

Supplement: Supplementary file 3 — Supplementary Figure1 [file 41419_2021_3865_MOESM3_ESM.png]

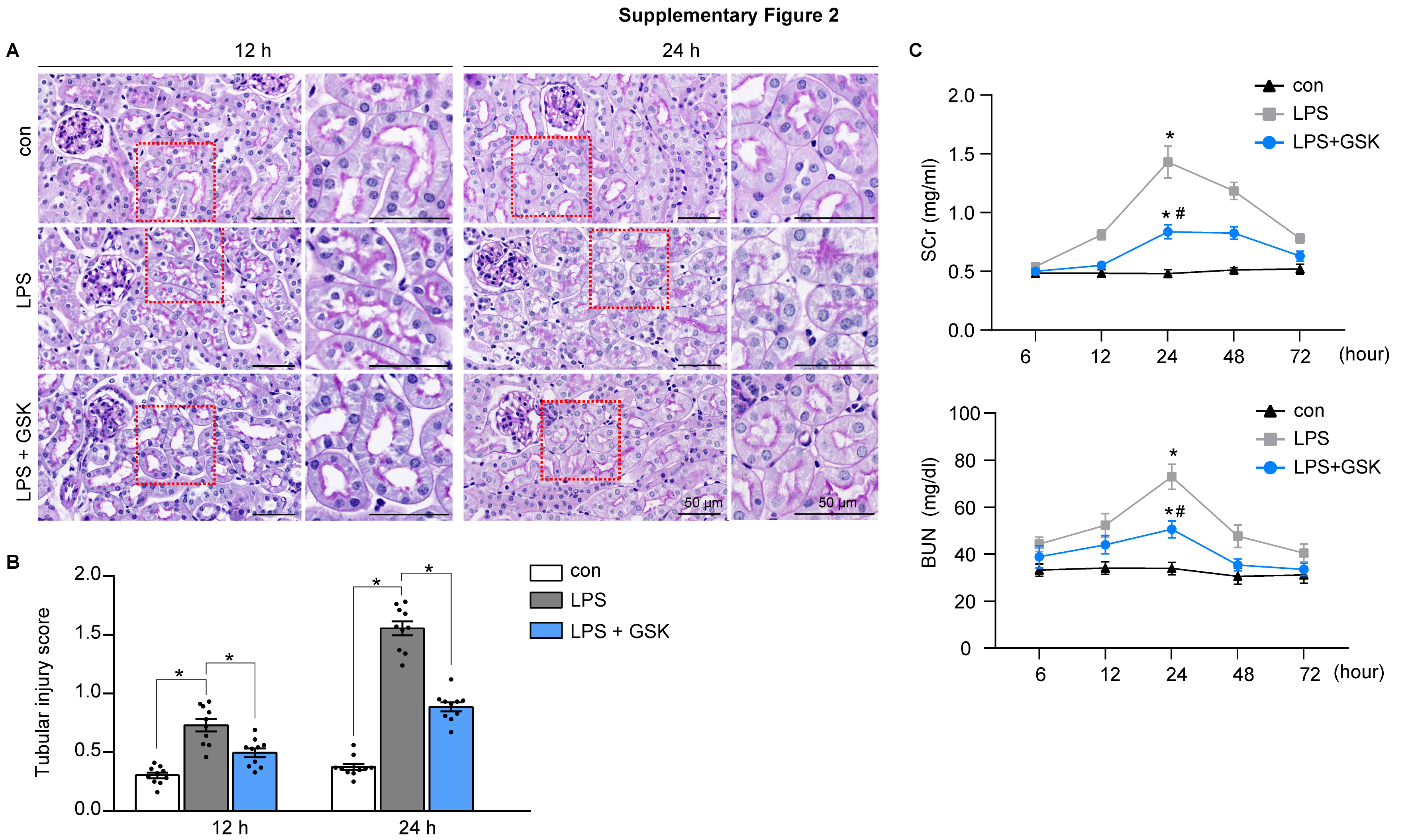

Supplement: Supplementary file 4 — Supplementary Figure 2 [file 41419_2021_3865_MOESM4_ESM.tif]

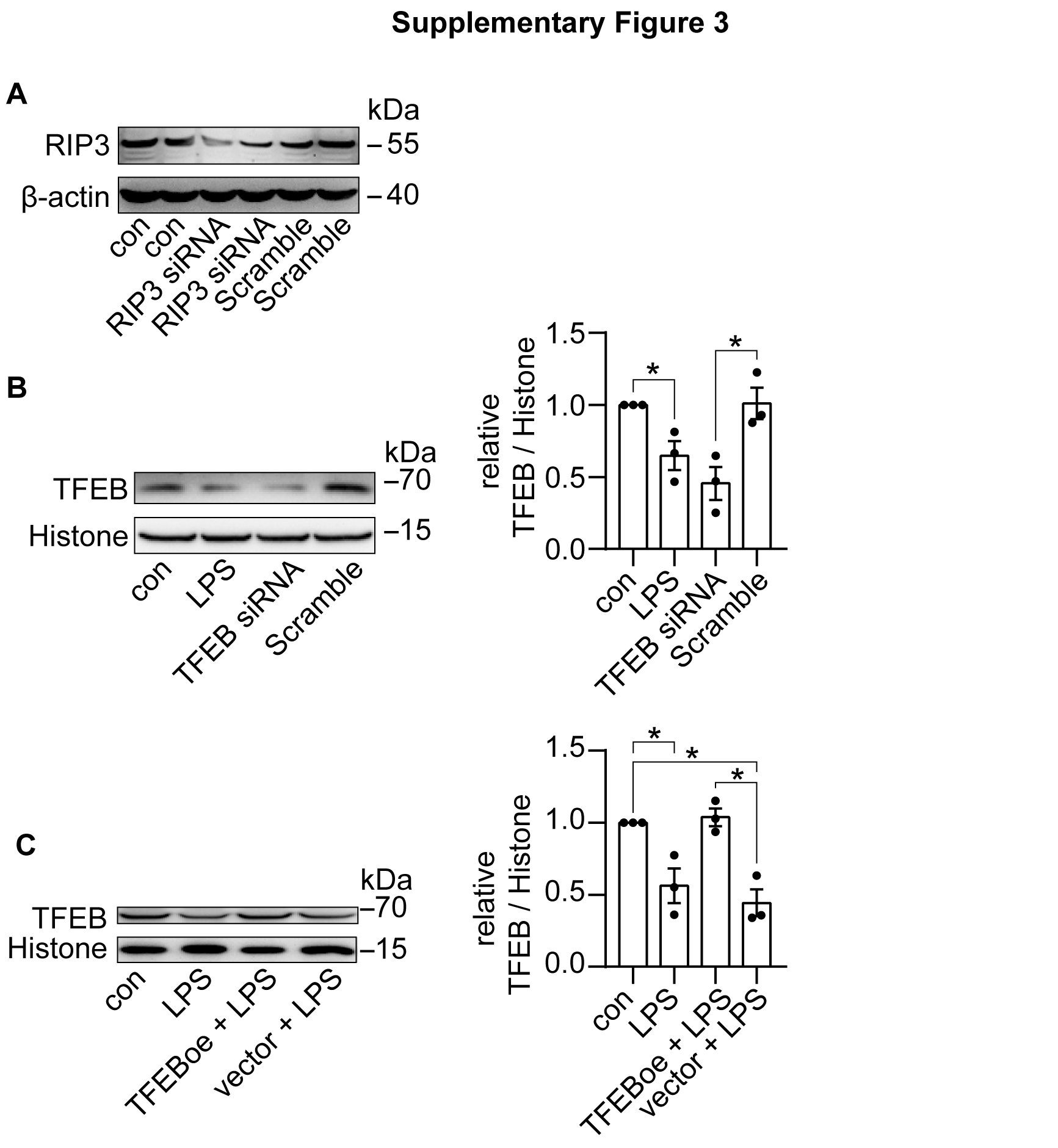

Supplement: Supplementary file 5 — Supplementary Figure 3 [file 41419_2021_3865_MOESM5_ESM.tif]

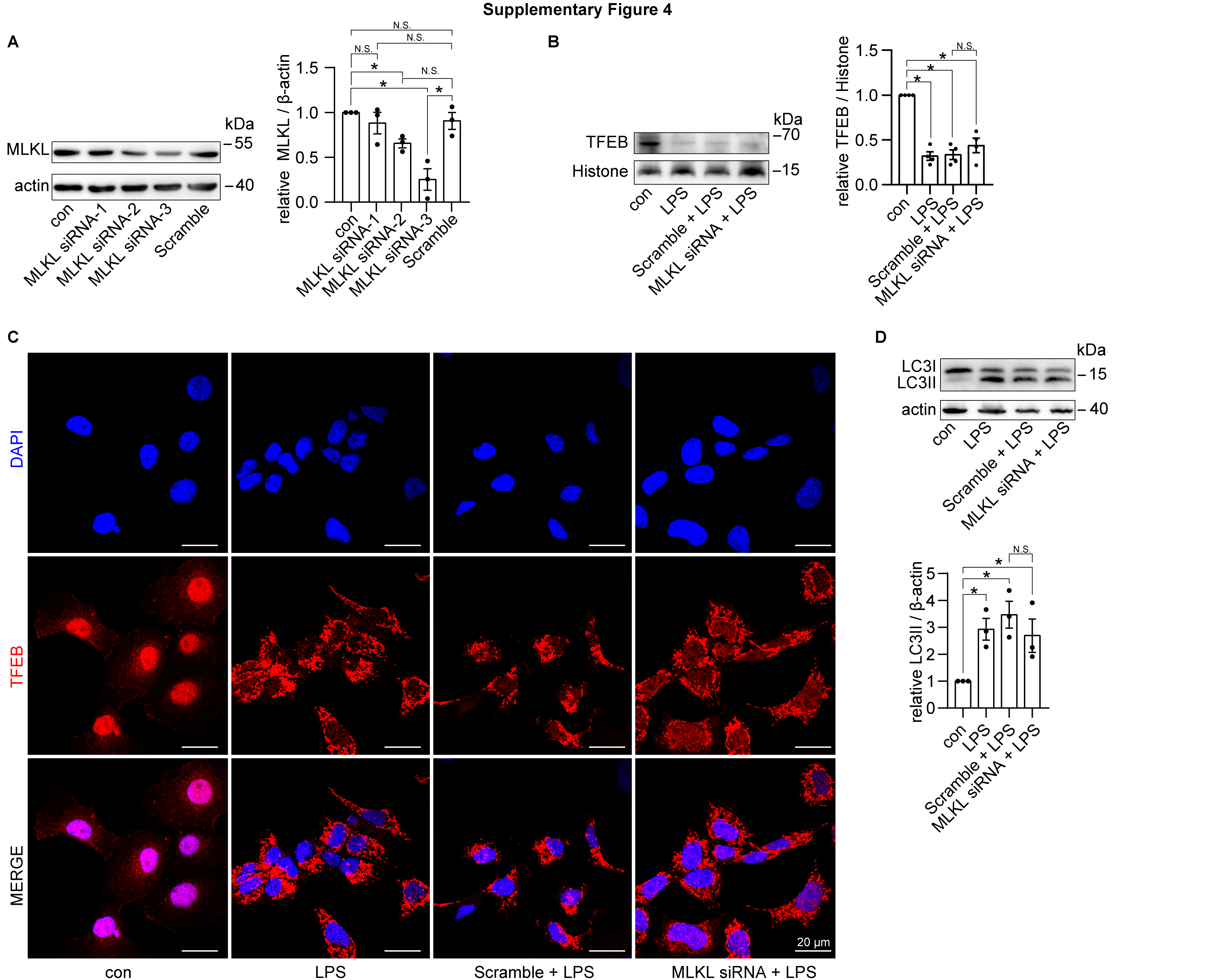

Supplement: Supplementary file 6 — Supplementary Figure 4 [file 41419_2021_3865_MOESM6_ESM.tif]
