# Supplementary material for: RIP3 impedes transcription factor EB to suppress autophagic degradation in septic acute kidney injury
Source: Cell Death Dis. 2021 Jun 8;12(6):593. doi: 10.1038/s41419-021-03865-8 (PMC8187512; doi:10.1038/s41419-021-03865-8)
Supplement: Supplementary file 7 — Supplementary Table 1 [file 41419_2021_3865_MOESM7_ESM.docx]

**Supplementary Table 1. Clinical characteristics in the patient with septic AKI and the control subject.**

|  | Gender | Date of birth | Body height(cm) | Body weight (kg) | Body temperature  (°F) | Scr  (μmol/l) | BUN (mmol/L) | WBC  (cell/L) | NEUT  （%） | PCT  (ng/ml) | CRP  (mg/L) | IL-6  (pg/ml) |
| --- | --- | --- | --- | --- | --- | --- | --- | --- | --- | --- | --- | --- |
| Septic AKI patient | male | 08/16/1996 | 172 | 65 | 103.1 | 1024 | 22.59 | 10.99*10^9^ | 64.9 | 21.02 | 36.31 | 13.1 |
| Control | male | 06/27/2000 | 166 | 65 | 97.5 | 88.26 | 5.76 | 7.39*10^9^ | 66.7 | - | 1.45 | - |

*serum creatinine: Scr; BUN: blood urea nitrogen; WBC: white blood cell count; NEUT: neutrophil ratio; PCT: procalcitonin;

°F: degree Fahrenheit; IL-6: Interleukin 6
