# Supplementary material for: RIP3 impedes transcription factor EB to suppress autophagic degradation in septic acute kidney injury
Source: Cell Death Dis. 2021 Jun 8;12(6):593. doi: 10.1038/s41419-021-03865-8 (PMC8187512; doi:10.1038/s41419-021-03865-8)
Supplement: Supplementary file 8 — Supplementary Table 2 [file 41419_2021_3865_MOESM8_ESM.docx]

**Supplementary Table 2. SiRNA sequences**

| Name | Sequence |
| --- | --- |
| RIPK3 siRNA | GCAGGAAATTTCAGGCCAA |
| TFEB siRNA | GACTCAGAAGCGAGAGCTA |
| MLKL siRNA-1 | CCTCTTCCATGAAGTGAAT |
| MLKL siRNA-2 | CCAACATCTTGCGTATATT |
| MLKL siRNA-3 | CCATTTGAAGGCTGTGATT |
