# Supplementary material for: RIP3 impedes transcription factor EB to suppress autophagic degradation in septic acute kidney injury
Source: Cell Death Dis. 2021 Jun 8;12(6):593. doi: 10.1038/s41419-021-03865-8 (PMC8187512; doi:10.1038/s41419-021-03865-8)
Supplement: Supplementary file 9 — Supplementary Table 3 [file 41419_2021_3865_MOESM9_ESM.docx]

**Supplementary Table 3. Primers used for RT-qPCR assays**

| Target | Forward primer (5'->3') | Reverse primer (5'->3') |
| --- | --- | --- |
| *GAPDH* | AGGTCGGTGTGAACGGATTTG | TGTAGACCATGTAGTTGAGGTCA |
| *Lc3b* | TTATAGAGCGATACAAGGGGGAG | CGCCGTCTGATTATCTTGATGAG |
| *p62* | AGGATGGGGACTTGGTTGC | TCACAGATCACATTGGGGTGC |
| *WIPI* | GGCTGTCTGTCAGGTGTTCCA | GCTCCCTGGTGTTCAGTATGG |
| *UVRAG* | TGCGATGGACTTTATCTGGTG | TGAGGCAAACTCTTCCTAACC |
| *VPS11* | AGGGTCGGTCAGCAGAGTCAA | TCCACGATCAGTTCCAACATC |
| *LAMP1* | CCAAACCTGTCACTGTCCACC | CCCACATTCAGCATCTCCAAC |
| *CathepsinB* | GGGAGGGATGGTGTATGGTAA | GACGGCTGTAATGGTGGCTAT |
