# Supplementary material for: RIP3 impedes transcription factor EB to suppress autophagic degradation in septic acute kidney injury
Source: Cell Death Dis. 2021 Jun 8;12(6):593. doi: 10.1038/s41419-021-03865-8 (PMC8187512; doi:10.1038/s41419-021-03865-8)
Supplement: Supplementary file 10 — Supplementary Table 4 [file 41419_2021_3865_MOESM10_ESM.docx]

**Supplementary Table 4. Primers used for ChIP assays**

| Target | Forward primer | Reverse primer |
| --- | --- | --- |
| *Lamp1* | GGGGTGGGGAGAGGGCAAGA | CCCGTGGACCGCCAGCTTAC |
| *CathepsinB* | CATCACGTGACGAATCAGCC | CAGCCACCAAGCCAAACG |
